# Preliminary Scales for *ICD-11* Personality Disorder: Self and Interpersonal Dysfunction Plus Five Personality Disorder Trait Domains

**DOI:** 10.3389/fpsyg.2021.668724

**Published:** 2021-07-12

**Authors:** Lee Anna Clark, Alejandro Corona-Espinosa, Shereen Khoo, Yuliya Kotelnikova, Holly F. Levin-Aspenson, Greg Serapio-García, David Watson

**Affiliations:** Department of Psychology, University of Notre Dame, Notre Dame, IN, United States

**Keywords:** *ICD-11* (International Classification of Diseases), personality disorder (PD), scale development, psychometric evaluation, assessment, personality functioning, personality traits

## Abstract

The *ICD-11* personality disorder model is the first fully dimensional assessment of personality pathology. It consists of a personality disorder (PD) dysfunction-severity dimension, which encompasses both self- and interpersonal dysfunction, and six optional qualifiers for five prominent personality traits—Negative Affectivity (NA), Detachment (DET), Dissociality (DSL), Disinhibition (DSN), and Anankastia (ANK)—plus a borderline pattern that is defined by the criteria of *DSM-IV* borderline PD. This article reports on the development of a new self-report measure to assess self- and interpersonal dysfunction and the five trait qualifiers. It is the first comprehensive measure of the *ICD-11* PD model in that (a) it is the only one to include both PD dysfunction-severity as well as trait scales and because (b) it is based on the Clinical Description and Diagnostic Guidelines, which are more detailed than the “statistical” model description that is currently on the *ICD-11* website. The authors wrote 992 items and then reduced the pool to 300 items by eliminating redundancy and selecting the consensus best few items for each subconstruct. Data were collected using an online sample of 383 Prolific workers. Using exploratory factor analysis, seven domain scales were developed, each of which contained two to four scales assessing components of the domain. These preliminary scales’ psychometrics were excellent, as were the domains’ and their components’ convergent and discriminant validity, with a few generally minor exceptions. Structural analyses at the component level revealed a three-factor structure consisting of two moderately correlated Internalizing factors, one centered on Self Dysfunction with two NA components and a DSN component (Distractibility) and the other on Interpersonal Dysfunction with DET and ANK components; as well as an Externalizing factor with DSL and a DSN component (Reckless Impulsivity) that was uncorrelated with the other two factors. Two aspects of the results in particular are striking: (1) ANK was not the opposite end of a DSN dimension, but rather contributed to an Internalizing Interpersonal Dysfunction dimension and (2) DSN had both an Internalizing and an Externalizing component. Implications of the findings and study limitations are discussed.

## Introduction

### Diagnosis of Personality Disorder in the ICD-11

In May of 2019, the World Health Organization (WHO) published the eleventh edition of the International Classification of Diseases (*ICD-11*), which contained the first fully dimensional model for the diagnosis of personality pathology. The new version abandoned the nine specific personality disorders included in *ICD-10* and replaced them with simply “personality disorder” (PD), characterized by “problems in functioning of aspects of the *self* (e.g., identity, accuracy of self-view), and/or interpersonal dysfunction (e.g., desire and ability to develop and maintain close and mutually satisfying relationships, ability to understand others’ perspectives, and to manage conflict in relationships) that have persisted over an extended period of time” (World Health Organization, 2020d). The disorder can be diagnosed at three levels of severity (mild, moderate, severe; plus a “severity unspecified” option), as well as described in more detail using a set of qualifiers “to describe the characteristics of the individual’s personality that are most prominent.” The qualifiers are five personality trait dimensions— Negative Affectivity (NA), Detachment (DET), Dissociality (DSL), Disinhibition (DSN), and Anankastia (ANK), basic definitions of which are available on the WHO website—and a Borderline pattern (World Health Organization, 2020e) that is based directly on borderline PD in the *Diagnostic and Statistical Manual, 4th Edition* (*DSM-IV*; American Psychiatric Association, 1994), the criteria for which were reproduced verbatim in Section II of *DSM-5* (American Psychiatric Association, 2013).

Personality disorder in *ICD-11* shares many characteristics with the Alternative Model of Personality Disorders (AMPD) in Section III of *DSM-5*, but also differs in several ways. Most importantly, the AMPD is a hybrid dimensional-categorical model in contrast to the ICD’s purely dimensional one. Specifically, although the two models share a dimensional core requirement of impairment in personality—self and/or interpersonal—functioning, and both have a dimensional trait system for further specification, the AMPD requires the latter, whereas the *ICD-11* trait and pattern qualifiers are optional. Moreover, the AMPD defines six specific PDs that are the primary diagnoses of the model. These categories are retained from earlier *DSM* editions, albeit they are now diagnosed using personality impairment and pathological trait dimensions rather than specific criteria. Moreover, the manual dictates: “Individuals who have a pattern of impairment in personality functioning and maladaptive traits that matches one of the six defined personality disorders should be diagnosed with that personality disorder” (p. 771).

A seventh AMPD diagnosis, PD-Trait Specified (PD-TS), is the closest counterpart to the *ICD-11* PD diagnosis. The PD-TS diagnosis “allows clinicians to tailor the description of each individual’s personality disorder profile, considering all five broad domains of personality trait variation and drawing on the descriptive features of these domains as needed to characterize the individual” (p. 770). Again, however, the *DSM-5* dictates that PD-TS should be used only if an individual’s “personality functioning or trait pattern is substantially different from that of any of the six specific personality disorders” (p. 771) whereas in *ICD-11*, a PD diagnosis is essentially the same as PD-TS.

Unlike the DSM, the *ICD-11* is published in several versions. As of this writing, the version on the WHO website is the “statistical” version, which provides the codes for WHO member-states to use for reporting health statistics as required by international agreement. In addition to the codes, the “Mental and Behavioral Disorder” chapter provides a general description of PD and of each of the five PD trait specifiers plus the borderline pattern specifier (World Health Organization, 2020d,e).

For general clinical, educational, and service use, the WHO develops “Clinical Descriptions and Diagnostic Guidelines” (CDDG) that describe “the main clinical and associated features of each mental-disorder category, followed by more operationalized diagnostic guidelines to assist clinicians in making diagnoses” (Clark et al., 2017, p. 80). Even so, the CDDG provides flexible guidance, that is, more prototypic descriptions than the DSM with its lists of specific criteria, and more formalized subcomponents of disorder definitions. The CDDG are published later than the statistical version to conduct “feasibility” studies to ensure that they are sufficiently clear and detailed to be useful for their intended purpose. As of this writing, the CDDG are being used in training sessions and these feasibility studies are ongoing.

The current study used the CDDG as the basis for writing items to assess the core construct and the trait specifiers^1^. We used the CDDG’s content components to guide item writing for both personality dysfunction and the trait specifiers. Although these components are akin to facets in the AMPD, they differ considerably in that they are not specific terms with formal definitions, but rationally based narrative paragraphs with content subcomponents, which can be somewhat overlapping. For example, the phrases “putting oneself and others at physical risk” and “put them or others in physical danger” appear as separate subcomponents of trait DSN. Nonetheless, for the purpose of organizing item writing, the first author developed an outline based on the CDDG, which is provided in Supplementary Table 1.

### Existing Measures of the ICD-11 Personality Disorder Model

When *DSM-5* was published, the AMPD included a clinician rating form to assess personality dysfunction, and a trait measure—the Personality Inventory for *DSM-5*—was included in both self-report and informant formats; however, no parallel instruments were developed in conjunction with the *ICD-11* PD model. To fill this gap, a number of measures of the *ICD-11* model have emerged. Two of these measures assess the only required element of the model: a designation of whether the personality disorder is mild, moderate, or severe. The Standardized Assessment of Severity of Personality Disorder (SASPD; Olajide et al., 2018), which was adapted from the Standardized Assessment of Personality–Abbreviated Scale (Moran et al., 2003), assesses PD severity via level of disability (minimal to none, mild, moderate, and severe) in nine areas of personality and psychosocial functioning—four that have an interpersonal focus (i.e., friendships; and being with, trusting, and caring about other people) and five that are more intrapsychic/behavioral (i.e., two items concerning affect and/or emotion regulation and three items assessing acting on impulse, being organized, and self-reliance, respectively). In contrast, Bach et al. (2021) developed the *ICD-11* Personality Disorder Severity Scale (PDS-*ICD-11*), a 14-item measure to assess aspects of personality functioning that contribute to *ICD-11* PD severity, based on the *ICD-11* CDDG, which include four aspects of self dysfunction (e.g., ability to maintain an overall positive and stable sense of self-worth), four aspects of interpersonal dysfunction (e.g., interest in engaging in relations with others), three domains in which personality dysfunction is manifested (i.e., emotional, cognitive, and behavioral) and, finally, the extent to which the dysfunction is associated with distress or disability in important areas of functioning (e.g., social, occupational).

The nine other measures of which we are aware all assess the optional qualifiers, with all but one focused solely on the trait qualifiers. Oltmanns and Widiger developed two of the nine:

(1) The 60-item Personality Inventory for *ICD-11* (P*ICD-11*; Oltmanns and Widiger, 2018), was based on brief draft descriptions of the *ICD-11* trait qualifiers provided in Tyrer et al. (2015) which are similar, but not identical to those of the approved statistical version. This measure has been translated into multiple languages, and the Italian (Somma et al., 2020) and Spanish (Gutiérrez et al., 2021) versions have been published. It also has an informant version (Oltmanns and Widiger, 2021) that has been used to study clinician ratings (Bach et al., 2020a).
(2) Oltmanns and Widiger (2020) developed the 121-item Five-Factor Personality Inventory for *ICD-11* (FFiCD), a 20-facet measure of the *ICD-11* qualifiers, by selecting facets of the Five-Factor Model Personality Disorder Scales (FFMPD) (Widiger et al., 2012) to measure both the traits—based, again, on the brief, draft descriptions provided in Tyrer et al. (2015)—and the borderline pattern qualifier.

The remaining seven instruments were developed using existing measures of personality traits in the maladaptive range, including five versions based on the Personality Inventory for *DSM*-5 (PID-5; Krueger et al., 2012; see Supplementary Table 2):

(1) A 143-item measure developed in both Danish and English that uses 16 PID-5 trait facets (Bach et al., 2017). The measure also has been used in both a Brazilian Portuguese (Lugo et al., 2019) and a Persian (Lotfi et al., 2018) version.
(2) A 158-item measure (Sellbom et al., 2020), which added two PID-5 facets (Suspiciousness and Attention Seeking) to those of Bach et al. (2017), based on updates in the *ICD-11* trait descriptions.
(3) The 34-item PID-5-Brief Form-Plus (PID-5-BF+, Kerber et al., 2020; most unfortunately titled because it is not based on the official 25-item PID-5-BF [American Psychiatric Association, 2013], with which it has only 8 items in common), which assesses the *ICD-11* PD model using two items each from 17 facets of the PID-5.
(4) A 36-item modified version of the PID-5-BF+ (Bach et al., 2020b), which assesses the *ICD-11* PD model using 18 PID-5 facets (and also the Psychoticism domain of the AMPD by adding its three PID-5 facets), differing from the Kerber et al. measure in that it omits the Perseveration facet from ANK and adds facets Orderliness and Rigidity, each also containing two items. This version was developed by an international group and has 12 different versions in various European languages plus Brazilian Portuguese.
(5) Bach and El Abiddine (2020) translated American Psychiatric Association (2013) 25-item PID-5-BF into Algerian to assess both models in a sample of Algerian college students. In broad outline, all five domains of the *ICD-11* PD model and also AMPD Psychoticism could be found, but a number of items did not perform as expected. It is unknown whether these represent cultural differences or translation-based problems, indicate that the PID-5 facets they tap are not central to their respective domains, or some combination of these factors. The results are consistent with some, but not all, other studies, so further research is needed.

Finally, Tyrer (2017) developed a 40-item measure based on the Personality Assessment Schedule (PAS; Tyrer and Alexander, 1979), the PAS *ICD-11*. This measure was then used as the basis for developing a 17-item, self-report, Korean version, the Personality Assessment Questionnaire for *ICD-11* (PAQ-11; Kim et al., 2020).

### Rationale for a New Measure of the ICD-11 Personality Disorder Model

Given this proliferation of measures of the *ICD-11* personality disorder model, it is reasonable to question the added value of yet another measure. However, there are three interrelated aspects of the preliminary measure presented in this article that collectively provide the rationale for its development. Specifically, the measure’s items were written (1) expressly to tap the *ICD-11* PD model, (2) based on the CDDG, and (3) to assess both PD severity and the five trait qualifiers. We discuss each of these in turn.

First, that the items were written expressly to tap the *ICD-11* PD model is a strength compared to measures using items that were developed for a similar, but not identical, model (e.g., the various measures that draw items from the PID-5 or that are based on the PAS). To be sure, there is a great deal of similarity between the AMPD and the *ICD-11* model of personality disorder, such that examining their interrelations is informative. At the same time, there are aspects of the AMPD that are not directly relevant to the *ICD-11* model (e.g., the specificity of the four Criterion A components and the 25 Criterion B facets), as well as vice versa. For example, personality disturbance in *ICD-11* is intended to reflect a single overarching dimension of impairment (which does not mean that it does not have various aspects to consider in making an overall determination of severity; e.g., Crawford et al., 2011; Tyrer et al., 2011; Olajide et al., 2018). In contrast, there remains considerable debate regarding whether AMPD Criterion A is similarly intended to reflect a single dimension (Morey, 2017; Hopwood et al., 2018), or a hierarchical model with either one (self and interpersonal) or two lower levels, with the self- and interpersonal-dysfunction components each breaking down into two subcomponents-respectively, identity and self-direction, and empathy and intimacy (see Sleep et al., 2019, for a discussion).

Perhaps the most critical limitation of measures based on the PID-5 is that the AMPD conceptualizes ANK as the opposite end of the DSN domain, and thus has limited content to assess the ANK domain, whereas in the *ICD-11* PD model, ANK is a distinct dimension that is theoretically unrelated to DSN (see McCabe and Widiger, 2020a, for a discussion of this issue). Accordingly, for the measure described in this article, care was taken to ensure that items written to assess these two domains were based on theoretically independent descriptions. We also aimed to make them empirically independent; for example, in developing the scales, items that distinguished the dimensions were preferenced over those that did not.

The second strength of the measure described in this article is that it is the only instrument to date that is based on the *ICD-11* PD CDDG descriptions of both PD severity and the trait qualifiers. This is advantageous because the CDDG are more extensive than the descriptions in the statistical version currently on the WHO website and more up-to-date than the descriptions in Tyrer et al. (2015). In particular, the descriptions are sufficiently elaborated that they can be subdivided into components for which subscales can be developed and tested for their coherence within the broader domains. Although a brief, domain-focused measure eventually will be needed for many clinical purposes in which limited time and resources prohibit use of an extensive measure of the *ICD-11* PD model, the initial development of a more expansive measure for the purpose of explicating the essential nature of the domains is an important step in establishing its construct validity and also towards developing a briefer measure that has strong psychometric properties and validly reflects the key properties of the constructs it assesses.

Third, as mentioned, the measure was developed to assess both PD severity and the trait qualifiers, whereas all other measures that were developed for *ICD-11* to date assess only one of these two components, but not the other. This is a strength for, again, three reasons. First, given that the sole requirement for diagnosing PD in the *ICD-11* is a determination that there is mild, moderate, or severe impairment in personality functioning, it is not clear how measures that assess only the optional trait qualifiers would be used clinically. Second, there is empirical support both for (e.g., Hopwood et al., 2011) and against (e.g., Sleep et al., 2019, 2020) the need for a severity criterion in the AMPD—which shares many features with the *ICD-11* PD model—in which case theoretical considerations must also be considered. Sharp and Wall (2021) have recently argued eloquently from a theoretical perspective for the importance of the severity dimension (see also Pincus et al., 2020). Echoing Livesley and Jang (2005), they defined personality impairment as “a general adaptive failure of a subjective intrapsychic system needed to fulfill adult life tasks” (p. 1). Sharp and Wall (2021) discuss the AMPD Criterion A’s connections to psychodynamic perspectives, but lest this aspect of the AMPD be a concern for any “hard-core empirical” PD researchers, we hasten to note that Livesley and Jang’s formulation was based on Allport (1937); Cantor (1990), and Plutchik (1980), particularly Plutchik’s evolutionary perspective, *not* on the psychodynamic literature. Co-development of measures of both major aspects of the *ICD-11* PD model provides an opportunity to consider theoretical issues regarding relations between them as part of the measure-development process (cf. Loevinger, 1957).

Third, it is well known that there is considerable overlap—both theoretical and empirical—between the general PD-severity dimension and personality trait dimensions that span the adaptive-maladaptive range (e.g., Widiger et al., 2019). However, *ICD-11* measures that were developed to assess only the general severity dimension or only the trait and pattern qualifiers were not able to consider the interrelatedness of these two diagnostic components in the development process. As a result they presumably did not ask such questions as—to use Allport’s terminology—do our item sets appropriately distinguish between what personality *is* (i.e., describe more stable and situation-general characteristics) and what personality *does* (i.e., assess how the person functions in the world)? In contrast, an important advantage of co-developing scales to assess these two aspects of personality pathology is that it facilitates study of the areas of overlap between them before the measures have been finalized, providing an opportunity to consider the “ideal” level of overlap from a theoretical perspective and potentially to shape the measures accordingly. To provide a specific example, particular aspects of interpersonal dysfunction may be more likely to overlap with DET and other aspects with DSL. If the scales are developed as part of the same measure, this can be studied directly and addressed within the scale development process, for example, by considering the balance between these two components of interpersonal dysfunction in the measure.

To be sure, a number of scales have been developed to assess the AMPD’s Criterion A and there are studies of their overlap with AMPD trait measures (e.g., Few et al., 2013; Hopwood et al., 2018; Sleep et al., 2019, 2020; McCabe and Widiger, 2020b), as well as parallel studies of *ICD-11* PD-severity measures and trait qualifiers (e.g., McCabe and Widiger, 2020a,b; Gutiérrez et al., 2021). These studies provide important information on existing scales, but their authors have little to no allowance for revising the measures to address problems that the studies reveal. To reiterate, the current measure is the only one that was developed expressly to assess directly both the *ICD-11* PD model’s primary dimension of PD severity and the trait qualifiers, as described fully in the CDDG, including consideration of their interrelations.^2^

In addition to these three main points (and their subpoints), a final strength of the current measure was the linguistic diversity of the item-development team. Besides five native speakers of English, the eight-person team^3^ included a native speaker of Spanish, Russian, and Malay, respectively. Because the *ICD-11* is used around the world, the translatability of items into each of these languages was an important consideration in item selection. Although these four languages represent a tiny subsample of world languages, they are from different language families, and thus provide a good starting point for developing an internationally useful measure.

## Method

### Participants and Procedure

All study procedures were approved by the Institutional Review Board of the University of Notre Dame. Participants were 383 community adults recruited via Prolific, an online crowdsourcing site. Eligibility requirements included at least 18 years of age, residence in an English-speaking country, and current or past history of mental-illness treatment. Participants gave informed consent as part of the online protocol.

#### Sample Characteristics

Participants were mostly from the United Kingdom (60%) or the United States (31%), and English was the native language of 97.4% of participants. Only 9 (2.3%) participants did not specify gender; of those who did, 52.1% identified as female and 47.9% as male. Mean age was 32.9 (*SD* = 11.3). Fifteen participants (5.8%) did not specify race, ethnicity, or both; of those who did, 92.7% identified as White, and the remainder as Asian (4.3%), Black (2.2%), or American Indian (< 1%); 3.2% identified as Hispanic. Despite the eligibility requirement, 22.4% reported no history of mental-health treatment; 56.8% had a past history only and 20.8% were currently in treatment, 1.3% for the first time. The eligibility criteria are controlled by Prolific, so the reason for the discrepancy between participants’ responses to Prolific’s and our question regarding history of mental-health treatment is unknown. One possibility is that the questions were phrased in different ways such that they could be answered differently for legitimate reasons; others are that it represents misrepresentation in one or the other context, random error, or a mix of these factors. A quarter (24.7%) of the sample were students, 35.5% were employed full-time and 22.7% part-time.

### Preliminary Scale Development Process

#### Definition of Constructs

As mentioned earlier, we used the CDDG to guide item writing. Specifically, the first author used the rationally based, narrative, prototypic descriptions of personality dysfunction and trait domains to develop structured, more formalized subcomponents of disorder definitions. Because the measure described herein is based on empirical responses to a particular set of items, items’ final organization may differ somewhat from that of the CDDG. Finally, we decided not to write items to assess the borderline pattern specifier because the description of that construct is simply a listing of the *DSM-IV* (and, therefore, *DSM-5*, Section II) criteria, for which multiple measures already exist.

#### Item Pool Development

Eight members of the Center for Advanced Measurement of Personality and Psychopathology (CAMPP), including both lab Directors, a postdoctoral scholar, three advanced graduate students, and two advanced undergraduate students met regularly to develop the item pool. Meetings were used to discuss construct—including component and subcomponent—definitions, and to select items from larger pools that had been written by rotating pairs of lab members between meetings. Using the outline derived by the first author from the *ICD-11* PD CDDG, items were written for each component/subcomponent of the PD-severity dimension and those of the five trait domains, yielding an initial pool of 992 items, which was then reduced by group selection of the preferred option among highly similar items. Finally, team members rated items within the various subcomponents of the dimensions and the top-rated items in each area were selected for inclusion. The final test pool was 300 items, ranging from 33 items for Self Dysfunction to 52 items for each of DET and DSL.

#### Data Collection

After indicating informed consent, participants were asked to provide demographic data and to rate how well each of the 300 items described them, using a 4-point scale ranging from Very or often False to Very or often True. The item set was administered together with items for an unrelated project for efficiency, so we report only on the *ICD-11* relevant measure. Participants were compensated for their time (averaging ∼30 min) and effort using Prolific’s recommended pay scale (which was $3.75 in this case). Of the 421 individuals who completed the study, 383 (91%) passed all four validity checks and provided sufficient data (< 5% missing) to impute. Validity checks consisted of items instructing participants to perform a certain action (e.g., Rate this item “Very or Often True”) or for which the correct answer is obvious (e.g., “I swim across the Atlantic Ocean every day”). Imputation was done at the item level using SAS Proc MI.

## Results

### Data Analytic Strategy

The primary goal of data analysis was to create preliminary scales for further testing and development in additional samples. We also wanted to examine the psychometric structure of the *ICD-11* model to determine its viability—specifically whether five distinct PD trait domains would emerge and also be relatively distinct from the two PD-severity dimensions, which were expected to be moderately highly correlated, consistent with their reflecting an overall dimension of PD severity.

For each domain, interitem correlations were examined, and if two or more items correlated ≥ 0.70, one was selected to represent their common content. An exploratory one-factor principal factor analysis (PFA) was then run on the items of each domain, and items that loaded < 0.30 were flagged for potential removal. Component scales were created for each empirical subdomain and examined for internal consistency. Items correlating < 0.30 with other items in the same component were flagged for potential removal.

Next, PFAs with promax rotation were run, extracting a successive number of factors until reaching either (a) the point at which there was an “elbow” in the plot of the eigenvalues or (b) fewer than 4 items defined the factor. These analyses indicated there were two primary components for each domain, with two exceptions: (1) For self-pathology, two of the four components emerged clearly, whereas the other two components’ items intermixed in two factors that were distinguished by keyed direction. Neither the two- nor three-factor solution provided more clarity, so as an interim solution, the rational component scales were retained. (2) For negative affectivity, a similar situation obtained, with two of four component scales emerging clearly and the other two intermixing on two separate factors. However, these components correlated ∼0.80, so they were combined. Factor analyses were then run on the items of each component to ensure that they formed a single factor, which they did. Finally, weaker items—those with loadings < 0.30 in both their respective domain and component scale analyses were removed. Table 1 lists the preliminary components for each domain, and, where applicable, their rationally based content areas.

**TABLE 1:** Preliminary empirically derived domain and component scales of the ICD-11 personality disorder model.

Domain	Component (# items)	Sample content
Self Dysfunction (30)	Identity Problems (8)	Puzzled by own behavior
	Low Self-accuracy (7)	Often misjudge own abilities
	Low Self-worth (8)	Difficulty maintaining positive self-image
	Low Self-direction (7)	Life lacks direction, goals
Interpersonal Dysfunction (35)	Relationship Difficulties (21)	Difficulty understanding others’ viewpoints
		Difficulty managing relationship conflict
		Difficulty developing/maintaining relationships
	Dysfunctional Engagement (14)	Little to no interest in developing relationships
		Will do anything to keep relationships from ending
		Think friends take too much time/effort
Negative Affectivity (30)	Emotional Lability (8)	Unpredictable emotions and moods; constant worry
	Negative Outlook (15)	View own life as a failure; expect the worst
	Mistrust (7)	Distrustful of others
Detachment (42)	Social Detachment (21)	Avoid social interactions; don’t enjoy social events
		Emotionally distant; keep to self even around others
		Have no close relationships
	Emotional Detachment (21)	Little reaction to positive or negative events
		Don’t get emotional; keep feelings to self
		Low or no interest in much of anything
Dissociality (46)	Low Empathy (30)	Deceive people to get what they wants
		No sympathy for others’ suffering
		Bully others; get violent easily
		Not bothered when hurt others or their feelings
Dissociality	Entitled Superiority (Self-centeredness) (16)	Expect special treatment; believe they deserve whatever is wanted
		Believe own needs more important than others’
		Believe oneself superior to others and that have done and/or will do great things
		Act to get attention; expect to be noticed, admired
Disinhibition (32)	Distractibility (19)	Easily distracted; struggle to stay on task
		Fail to complete tasks; don’t finish on time
		Like to keep options open; goals provide direction (R)
	Impulsive Recklessness (13)	Want desired things right away
		Don’t consider consequences; blurt things out
		Enjoy risky activities; don’t turn down chances for fun
Anankastia (31)	Hypercontrol (19)	Avoid activities if outcome uncertain; hypercautious
		Weigh all possibilities before decision-making
		Refuse to change routine; others notice inflexibility
	Perfectionism (12)	Must meet all standards of perfection, especially own
		Work must be perfect in all details; expect same high standards of everyone
		Home must be perfectly neat, with everything in its proper place

**ICD-11*, International Classification of Diseases, 11^th^ Ed. (World Health Organization, 2020).*

### Psychometric Properties

#### Descriptive Statistics

Descriptive statistics for the seven domain scales and their component scales are shown in Table 2. Of the possible range from 1 to 4, domain-level means ranged from 1.62 (DSL) to 2.64 (NA), *M* = 2.32, slightly below the midpoint of the range. Standard deviations were all within a small range (0.40 to 0.62; *M* = 0.51); indicating similar within-scale variability across domains. Within domains, participant scores ranged from 1 to 3.93 (of 4); the smallest range was 1.85 (DSL; from 1 to 2.85) and the largest was 2.91 (DET; from 1.02 to 3.93), *M* = 2.42. Similarly, component-level means ranged from 1.55 (Entitled Superiority) to 3.02 (Low Self-worth), *M* = 2.36. Participant scores ranged from 1 to 4 on five component scales, all of which were from either Self Dysfunction or NA, indicating that the full range of possible scores were represented in these domains. Interpersonal Dysfunction’s Relationship Difficulties scale and the two DSL component scales had the lowest maximum values (3.44 to 3.48), and the former had the smallest range (2.43), but even that is 81% of the possible range, indicating that the sample’s personality impairment and maladaptive trait levels were generally broadly diverse.

**TABLE 2:** Descriptive statistics for the domain and component scales of the ICD-11 PD model.

Scale	# items	Mean	*SD*	*Range*	*Omega*	*AIC*
Self Dysfunction	30	2.60	0.46	1.13 – 3.73	0.91	0.25
Identity Problems	8	2.41	0.58	1.00 – 4.00	0.80	0.33
Low Self-worth	8	3.02	0.62	1.00 – 4.00	0.85	0.41
Low Self-accuracy	7	2.40	0.48	1.00 – 3.57	0.72	0.27
Low Self-directedness	7	2.56	0.62	1.00 – 4.00	0.83	0.39
Interpersonal						
Dysfunction	35	2.22	0.41	1.29 – 3.29	0.90	0.20
Relationship Difficulties	21	2.26	0.47	1.05 – 3.48	0.88	0.26
Dysfunctional Engagement	14	2.15	0.50	1.00 – 3.86	0.86	0.29
Negative Affectivity	30	2.64	0.49	1.00 – 3.73	0.92	0.28
Negative Outlook	15	2.51	0.58	1.00 – 3.87	0.89	0.35
Emotional Lability	10	2.85	0.54	1.00 – 4.00	0.83	0.33
Mistrust	5	2.58	0.61	1.00 – 4.00	0.79	0.43
Detachment	42	2.45	0.53	1.02 – 3.93	0.95	0.31
Social Detachment	21	2.62	0.60	1.05 – 3.95	0.94	0.43
Emotional Detachment	21	2.27	0.57	1.00 – 3.90	0.92	0.35
Dissociality	46	1.62	0.40	1.00 – 2.85	0.94	0.25
Low Empathy	30	1.66	0.44	1.00 – 3.47	0.93	0.31
Entitled Superiority	16	1.55	0.48	1.00 – 3.44	0.91	0.36
Disinhibition	32	2.25	0.43	1.28 – 3.66	0.89	0.23
Distractibility	19	2.50	0.54	1.05 – 3.89	0.91	0.35
Reckless Impulsivity	13	1.88	0.49	1.08 – 3.62	0.83	0.26
Anankastia	31	2.45	0.44	1.26 – 3.74	0.90	0.23
Hypercontrol	19	2.46	0.46	1.21 – 3.74	0.87	0.26
Perfectionism	12	2.42	0.57	1.08 – 3.83	0.86	0.34

**N* = 383 community adults. *ICD-11*, International Classification of Diseases, 11^th^ Ed. (World Health Organization, 2020); PD, Personality Disorder; *SD*, Standard deviation. AIC, Average interitem correlation.*

Internal consistency estimates (McDonald’s omega) for the domain scales ranged from 0.89 (32-item DSN) to 0.95 (42-item DET); median = 0.91. For the component scales, the omega range was from 0.72 (7-item Low Self-accuracy) to 0.94 (21-item Social Detachment and 30-item Low Empathy); median = 0.86. The only other component scale with omega < 0.80 was 5-item NA Mistrust (0.79). For the domain scales, average interitem correlations (AICs) ranged from 0.20 (35-item Interpersonal Dysfunction) to 0.31 (42-item DET); *M* = 0.25, indicating that the domain scales assess coherent, but relatively broad constructs. For the component scales, the AIC range was from 0.26 (13-item Reckless Impulsivity and 19-item Hypercontrol) to 0.43 (5-item Mistrust and 21-item Social Detachment), *M* = 0.34. In all cases, the component scales’ AICs were higher than their respective domain scales’ AICs, indicating that the component scales assess narrower constructs than their corresponding domain scales, although the differences were quite small in a few cases (e.g., Self-dysfunction’s AIC was 0.25 and its component Low Self-accuracy’s AIC was 0.27). Overall, domain and component scales had good to excellent internal consistency reliability.

### Interscale Correlations

#### Domains

Correlations among the seven domain scales are shown in Table 3. The self- and interpersonal pathology domain scales correlated 0.45, which is fairly close to the mid-point of the range seen in existing AMPD measures of these constructs. For example, in separate community adult and undergraduate samples, Corona-Espinosa et al. (2021) found that interscale correlations among the subscales of six measures of AMPD Criterion A ranged from 0.34 to 0.75 (median = 0.53). The wide range of correlations reflects ongoing theoretical debate regarding the degree to which self- and interpersonal dysfunction are correlated versus distinct constructs.

**TABLE 3:** ICD-11 personality disorder-severity and trait domain scale intercorrelations.

	1	2	3	4	5	6	7
*Functioning Domains*							
1. Self Dysfunction	(0.91)						
2. Interpersonal Dysfunction	**0.45**	(0.90)					
*Trait Domains*							
3. Negative Affectivity	**0.83**	**0.58**	(0.92)				
4. Detachment	**0.45**	**0.77**	**0.58**	(0.95)			
5. Dissociality	−0.01	0.*32*	0.12	0.14	(0.94)		
6. Disinhibition	**0.57**	0.*38*	**0.49**	0.29	0.*37*	(0.89)	
7. Anankastia	0.25	0.*33*	**0.44**	0.*35*	0.06	−0.04	(0.90)

**N* = 383 community adults. *ICD-11*, International Classification of Diseases, 11^th^ Ed. (World Health Organization, 2020). McDonald’s omega are in the diagonal. Correlations ≥ 0.40 are **bolded**; those < 0.40 and ≥ 0.30 are *italicized.**

Correlations among the PD trait-domain scales ranged from −0.04 between DSN and ANK to 0.58 between NA and DET (overall mean |*r*|^4^ = 0.30). This is comparable to the mean 0.31 correlation reported by Oltmanns and Widiger (2018) for their PiCD, and lower than the mean 0.43 correlation reported by Bach et al. (2018) among *ICD-11* PD trait domain scores using the 16-facet Bach et al. (2017) measure. However, the convergent/discriminant pattern differed across measures. Specifically, in the current measure, three |*r*| s were > 0.40, including one > 0.50, and all involved NA; other |*r*| s were all ≤ 0.37 and only DSL had no |*r*| ≥ 0.40. In Oltmanns and Widiger (2018), four |*r*| s were ≥ 0.40 and none were ≥ 0.50; other |*r*| s were all ≤ 0.32) and all scales had at least one |*r*| > 0.40. In Bach et al. (2018), seven of the 10 |*r*| s were ≥ 0.40, including one > 0.50; other |*r*| s were all < 0.40) and all scales had at least one |*r*| > 0.40. In any case, these results indicate that the current measure could be improved by revising the NA scale to lower its higher discriminant correlations.

Turning to relations between the PD-severity and trait-qualifier dimensions, as shown in Table 4, the Self and Interpersonal Dysfunction scales both have strong relations with the trait-domain scales. Specifically, Self Dysfunction correlated moderately to strongly with three trait domains—NA (*r* = 0.83), followed by DSN (*r* = 0.57), and DET (*r* = 0.45)—whereas Interpersonal Dysfunction correlated strongly with DET (0.77) and moderately strongly with NA (0.58). In contrast, DSL correlated −0.01 and 0.32, and ANK correlated 0.25 and 0.33 with Self and Interpersonal Dysfunction, respectively. These relations are consistent with those reported by Clark and Ro (2014) using then-existing measures of PD severity and traits before either the *DSM-5* AMPD or *ICD-11* models as developed (see their Table 4). Because both the PD-severity and trait-domain scales have component scales, these data can be used to examine the nature of the overlap between these two aspects of personality pathology.

**TABLE 4:** ICD-11 personality disorder-severity component scales: Correlations among them and with PD-trait component scales.

	1	2	3	4	5	6
Personality-Severity Components						
1. SD Identity	(0.80)					
2. SD Low Self-worth	**0.51**	(0.85)				
3. SD Low Self-accuracy	**0.63**	**0.45**	(0.72)			
4. SD Low Self-directedness	**0.61**	**0.53**	**0.46**	(0.83)		
5. ID Relationship Difficulties	**0.50**	**0.43**	0*.39*	**0.45**	(0.88)	
6. ID Dysfunctional Engagement	0.16	0.10	−0.02	0.21	**0.46**	(0.86)
PD-Trait Components						
7. NA Negative Outlook	0.67*	0.75*†	0.53*	**0.73**	0.57*	0.21
8. NA Emotional Lability	**0.52**	**0.72**†****	**0.40**	**0.45**	0.58*	0.15
9. NA Mistrust	0*.34*	*0.37*	0.27	0*.32*	**0.56**†****	0*.39*
10. DET Social Detachment	0*.33*	**0.40**	0.15	**0.43**	0.58*	0.75*†
11. DET Emotional Detachment	0*.38*	0*.31*	0.22	**0.42**	**0.56**†****	**0.55**†****
12. DSL Low Empathy	0.16	−0.09	0.11	0.13	**0.41**†****	0.26
13. DSL Entitled Superiority	−0.12	−0.21	0.00	−0.23**†**	0.14	−0.07
14. DSN Distractibility	**0.50**	**0.41**	**0.41**	0.76*†	**0.47**	0.23
15. DSN Reckless Impulsivity	0.26	−0.10	0.29**†**	0.23	0.23	−0.06
16. ANK Hypercontrol	0.27	**0.47**†****	0.20	0.21	**0.40**	0.22
17. ANK Perfectionism	−0.01	0.18	0.00	−0.10	0.20**†**	0.06

*N* = 383 community adults. *ICD-11*, International Classification of Diseases, 11^th^ Ed. (World Health Organization, 2020); PD, Personality Disorder; SD, Self Dysfunction; ID, Interpersonal Dysfunction; NA, Negative Affectivity; DET, Detachment; DSL, Dissociality; DSN, Disinhibition; ANK, Anankastia. For the personality-severity components, McDonald’s omegas are in the diagonal and convergent (i.e., within-domain) correlations are underlined. Correlations ≥ 0.40 are **bolded**; those < 0.40 and ≥ 0.30 are italicized.*Highest correlation in each column. ^†^Highest correlation in each row.

#### Components

Table 4 presents correlations among the PD-severity component scales and also their correlations with the trait-component scales. Overall, the PD-severity component scales showed a good convergent-discriminant pattern, with the averages of the respective Self- and Interpersonal Dysfunction component scales each correlating significantly higher within subdomain (0.54 and 0.46, respectively) than across subdomains (0.29; all *p*s < 0.05). However, the correlation of Relationship Difficulties with Identity (0.50) was higher than its 0.46 correlation with Dysfunctional Engagement (the other Interpersonal Dysfunction component), as well as higher than two of the six correlations among the Self Dysfunction components, although none of these differences was statistically significant. Thus, 80% of the component scale correlations were consistent with expectation, which is promising, while also indicating room for improvement.

Regarding relations between the PD-severity and trait components, correlations ranged from −0.23 to 0.76 (*M* = 0.32); ignoring sign, the range was from 0.00 to 0.76 (*M* = 0.35), indicating a wide range of relations between these two broad aspects of personality pathology. Relationship Difficulties stood out as particularly strongly correlated across the two aspects of personality pathology: It correlated > 0.50 with all NA and DET components, as well as > 0.40 with Low Empathy, Distractibility, and Hypercontrol. Conversely, three PD-trait components correlated moderately to strongly with five of the six PD-severity components: Specifically, Negative Outlook, Emotional Lability, and Distractibility correlated > 0.40 with all PD-severity components except Dysfunctional Engagement, including four correlations > 0.70. We discuss the significance of these patterns later in the paper.

Finally, Table 5 presents correlations among the PD-trait components, which generally exhibited good convergent-discriminant patterns. Specifically, the mean convergent (i.e., within-domain) component correlations were higher than the mean discriminant (i.e., cross-domain) component correlations—with three notable exceptions: Two were the discriminant correlations of DSN’s Distractibility component with NA’s Negative Outlook and Emotional Lability components—*r*s = 0.63 and 0.46—which were significantly higher (*p* < 0.02) than its convergent correlation of 0.33 with DSN Reckless Impulsivity^5^. The Distractibility–Negative Outlook discriminant correlation was also significantly higher than Negative Outlook’s convergent correlation with Mistrust (*r* = 0.47); no other comparisons with the convergent correlations of these two NA components were significant.

**TABLE 5:** Intercorrelations among ICD-11 personality disorder PD-trait component scales.

		1	2	3	4	5	6	7	8	9	10	11
1. NA	Negative Outlook	0*.89*										
2. NA	Emotional Lability	***0.66***	0*.83*									
3. NA	Mistrust	***0.47***	***0.41***	0*.79*								
4. DET	Social Detachment	**0.51**	**0.40**	**0.53**	0*.94*							
5. DET	Emotional Detachment	**0.46**	**0.26**	**0.47**	***0.67***	0*.92*						
6. DSL	Low Empathy	0.15	0.16	0.29	0.19	0*.34*	0*.93*					
7. DSL	Entitled Superiority	−0.14	0.00	0.06	−0.20	−0.08	***0.51***	0*.91*				
8. DSN	Distractibility	**0.63**	**0.46**	0.28	0*.38*	*0.34*	0.24	−0.08	0*.91*			
9. DSN	Reckless Impulsivity	0.10	0.03	0.10	−0.11	0.07	**0.56**	**0.40**	*0.33*	0*.83*		
10. ANK	Hypercontrol	**0.51**	**0.49**	0*.33*	**0.46**	0*.38*	0.04	−0.08	0.21	−0.27	0*.87*	
11. ANK	Perfectionism	0.07	0.21	0.24	0.11	0.10	0.06	0.18	−0.09	−0.14	***0.50***	0*.86*

**N* = 383 community adults. *ICD-11*, International Classification of Diseases, 11^*th*^ Ed. (World Health Organization, 2020). NA, Negative Affectivity; DET, Detachment; DSL, Dissociality; DSN, Disinhibition; ANK, Anankastia. McDonald’s omegas are in the diagonal. Correlations > 0.40 are **bolded**; those < 0.40 and > 0.30 are *italicized.* Convergent correlations are underlined.*

The third exception was that the discriminant correlation between DSN’s Reckless Impulsivity component and DSL’s Low Empathy component (*r* = 0.56) was significantly higher than the former’s, but not the latter’s convergent correlation (*r*s = 0.33 and 0.51, respectively). Note that all of these unexpected outcomes involved DSN components, whose 0.33 correlation was surprisingly low. We discuss this issue later.

### Structural Analyses

#### Preliminary Analyses

Parallel analyses were run on the (1) PD-severity components, (2) PD-trait components, and (3) combined PD-severity and trait components and indicated two, three, and four factors respectively. Promax-rotated principal factors analyses were run on each set of component scales, extracting from one factor up to the maximum number of factors indicated by parallel analyses, and then examined for viability (e.g., number of marker variables) and interpretability. For the PD-severity analysis, the two-factor solution was optimally interpretable and is presented in the upper half of Table 6. For the PD-trait analysis, both the two- and three-factor solutions were interpretable, but one ANK component had a low loading on both factors in the two-factor solution, so the three-factor solution is presented in the lower half of Table 6.

**TABLE 6:** Promax-rotated principal axis factor analyses of ICD-11 PD-severity and PD-trait component scales, respectively.

			PD severity	
			
Domain			Self	Interpersonal
component			Dysfunction	Dysfunction
SD	Identity		**0.79**	0.02
SD	Low Self-accuracy		**0.79**	−0.18
SD	Low Self-directedness		**0.65**	0.13
SD	Low Self-worth		**0.63**	0.04
ID	Dysfunctional engagement		−0.12	**0.64**
ID	Relationship Difficulties		0*.36*	**0.50**

		**PD traits**		
		
		**Internalizing**	**Externalizing**	**Anankastia**

NA	Negative Outlook	**0.85**	−0.06	−0.07
DET	Social Detachment	**0.73**	−0.12	**0.10**
DSN	Distractibility	**0.70**	**0.08**	−*0.32*
DET	Emotional Detachment	**0.64**	0.08	**0.08**
NA	Emotional Lability	**0.63**	**0.02**	0.14
NA	Mistrust	**0.54**	0.17	**0.22**
DSL	Low Empathy	0.21	**0.74**	0.07
DSL	Entitled Superiority	−0.24	**0.69**	0.25
DSN	Reckless Impulsivity	0.10	**0.67**	−*0.30*
ANK	Perfectionism	0.01	0.12	**0.66**
ANK	Hypercontrol	**0.48**	−0.14	**0.54**

**N* = 383 community adults. *ICD-11*, International Classification of Diseases, 11^th^ Ed. (World Health Organization, 2020); PD, Personality Disorder; SD, Self Dysfunction; ID, Interpersonal Dysfunction; NA, Negative Affectivity; DET, Detachment; DSN, Disinhibition; DSL, Dissociality; ANK, Anankastia.*

*Factor loadings ≥ 0.40 are **bolded**; those < 0.40 and ≥ 0.30 are *italicized.* The two PD severity factors correlate 0.47, whereas the three PD trait factors’ correlations are 0.09 (Internalizing-Externalizing), 0.17 (Internalizing-Anankastia), −0.14 (Externalizing-Anankastia).*

For the combined analyses, the three-factor solution was again optimally interpretable and is presented in Table 7. An argument can be made for the four-factor solution, but several variables in this solution did not mark any factor at 0.40 or higher, plus the fourth factor was marked only by the two ANK components, so the solution seems overextracted. Therefore, the three-factor solution was selected. The results of all other solutions run are provided in Supplementary Tables 3–7.

**TABLE 7:** Promax-rotated three-factor principal axis factor analysis of ICD-11 personality disorder-severity and trait component scales.

		Internalizing	Internalizing	
Domain		Self	interpersonal	
component		Dysfunction	Dysfunction	Externalizing
SD	Self-directedness	**0.84**	−0.06	0.02
NA	Negative Outlook	**0.84**	0.14	−0.11
SD	Identity Problems	**0.79**	−0.05	0.06
DSN	Distractibility	**0.73**	−0.03	0.16
SD	Self-accuracy	**0.73**	−0.19	0.09
SD	Self-worth	**0.73**	0.10	−*0.36*
NA	Emotional Lability	**0.63**	0.19	−0.11
DET	Social Detachment	0.09	**0.82**	−0.07
ID	Dysfunctional Engagement	−0.19	**0.80**	0.11
DET	Emotional Detachment	0.14	**0.64**	0.13
ANK	Hypercontrol	0.18	**0.53**	−*0.30*
NA	Mistrust	0.20	**0.51**	0.12
ID	Relationship Difficulties	0*.37*	**0.50**	0.26
ANK	Perfectionism	−0.15	**0.41**	−0.09
DSL	Lack of Empathy	−0.02	0.29	**0.74**
DSN	Reckless Impulsivity	0*.31*	−0.27	**0.74**
DSL	Entitled Superiority	−0.20	0.04	**0.59**

**N* = 383 community adults. *ICD-11*, International Classification of Diseases, 11^th^ Ed. (World Health Organization, 2020); SD, Self Dysfunction; NA, Negative Affectivity; DSN, Disinhibition; DET, Detachment; ID, Interpersonal Dysfunction; ANK, Anankastia; DSL, Dissociality. Factor loadings ≥ 0.40 are **bolded**; those < 0.40 and ≥ 0.30 are *italicized.* The three factors’ correlations are 0.47 (Internalizing- Self and Interpersonal Dysfunction), 0.09 (Internalizing Self Dysfunction and Externalizing), and 0.04 (Internalizing Interpersonal Dysfunction and Externalizing).*

#### Structures of PD Severity and PD Traits

As shown in the upper portion of Table 6, the Self- and Interpersonal Dysfunction scales formed clear, distinct factors, with the only notable cross loading being 0.36 (Relationship Difficulties on Self Dysfunction). The factors correlated 0.47, indicating that together they reflect a higher order dimension of personality pathology.

The three factors of the PD-trait structure shown in the lower portion of Table 6 can be labeled Internalizing, Externalizing, and Anankastia. The first (Internalizing) factor consisted of the three NA components, both DET components, and DSN Distractibility, with a strong (0.48) cross-loading by ANK Hypercontrol. The second (Externalizing) factor consisted of both DSL components and DSN Reckless Impulsivity. Finally, the two ANK components loaded on the third factor, with moderate cross loadings of −0.32 and −0.30 by the two DSN components. Thus, as foreshadowed in the correlational analyses, DSN—whose components are only moderately correlated (*r* = 0.33)—contains both an Internalizing (Distractibility) and an Externalizing (Reckless Impulsivity) aspect, both of which cross load on the Anankastia factor. The three factors are largely independent of each other, with correlations of only 0.17 (Internalizing with Anankastia) 0.09 (Internalizing with Externalizing) and −0.14 (Externalizing with Anankastia).

#### Combined Structure of PD Severity and Traits

Finally, Table 7 shows the joint three-factor structure of PD severity and traits. It has two Internalizing-pathology dimensions—one each centered on Self Dysfunction and Interpersonal Dysfunction, respectively—and an Externalizing-pathology dimension. The Internalizing Self Dysfunction factor is marked by all four Self Dysfunction components, two of the three NA components (Negative Outlook and Emotional Lability), and DSN’s Distractibility, with cross-loadings of 0.37 and 0.31 by Interpersonal Dysfunction’s Relationship Difficulties component and the other DSN component, Reckless Impulsivity. The Internalizing Interpersonal Dysfunction factor is marked by all of the DET and Interpersonal Dysfunction components, NA’s Mistrust component, and ANK’s Hypercontrol component. Finally, the Externalizing factor is marked by both DSL components and DSN’s Reckless Impulsivity component, with cross-loadings of −0.36 and −0.30 by Self Dysfunction’s Low Self-worth and ANK’s Hypercontrol components, respectively.

The joint PD-severity and traits analysis yields a somewhat different trait organization from the separate analyses. Specifically, the NA and DET components load together in the three-factor trait structure but split into two factors in the three-factor combined severity-trait structure with two of the NA and all of the Self Dysfunction components factoring together and the DET and Interpersonal Dysfunction components factoring together. This makes sense because although the NA and DET components correlate moderately strongly (*M r* = 0.44), each is more strongly correlated with its respective PD-severity counterpart (*M r*s of NA–Self Dysfunction and of DET–Interpersonal Dysfunction components = 0.53 and 0.62, respectively).

It is noteworthy that co-factoring PD severity and traits also affects the inter-factor correlations: In the trait-only structure, the three factors are almost entirely independent, whereas in the combined structure the two Internalizing factors correlate 0.47—reflecting the interrelation of the two (Self- and Interpersonal) personality-functioning impairment domains, as well as the moderately strong correlations between the NA and DET component scales. Both Internalizing factors remained unrelated to the Externalizing factor (factor correlations were 0.09 and 0.04 with the Internalizing Self- and Interpersonal Dysfunction factors, respectively).

## Discussion

As far as we are aware, there are no structural studies of *ICD-11* PD severity or the joint structure of *ICD-11* PD-severity and PD traits to date, other than the current study, in part because existing *ICD-11* PD-severity measures are global measures that are not well suited for analyses of their components. Moreover, most studies of the trait-structure of the *ICD-11* PD model have been based largely on either the PID-5, which lacks an anankastic scale, or on early brief descriptions of the traits, which are less well elaborated than the CDDG. Thus, the only instruments with which the current measure is comparable assess the AMPD. To date, joint studies of AMPD Criterion A and B measures have revealed a high degree of overlap and based on the analyses presented herein, it appears that the same may be true of the *ICD-11* PD model, which we now discuss in more detail.

### Implications of Results

#### Basic Scale Properties

Two particular findings are noteworthy: First, the number of items per component scale ranged widely from 5 to 30 items, which suggests that either (a) the measure might benefit from further factoring of some of the longer scales or (2) the longer scales should be further pruned, which would result in a tighter measure with fewer items. It is arguably appropriate to pursue both approaches, with the first leading to a more differentiated structure that would facilitate research delving more deeply into the nature of the constructs, whereas the latter would be helpful from a clinical perspective, that is, yielding a shorter measure that would provide an overview of patients’ personality pathology.

Second, the scales’ psychometrics generally indicated that participants used almost the whole range of item ratings across scales. However, the maximum value on the DSL domain and component scales suggests that externalizing pathology may have been somewhat underrepresented in the sample. Conversely, that the full range of 1 to 4 was used for three Self Dysfunction component scales and two NA component scales suggests that internalizing pathology may have been relatively overrepresented. Thus, it will be important to examine the measure’s performance in samples with greater representation of externalizing relative to internalizing pathology.

#### Convergent and Discriminant Validity of PD-Severity Scales

Regarding convergent validity, the two functioning-severity scales correlated 0.45, indicating a shared higher order dimension with clearly distinct components. These data thus contribute to the debate regarding the extent to which personality pathology is a single dimension versus has a hierarchical structure (see Sleep et al., 2019, discussion) by adding support for a hierarchical conceptualization.

Second, considering the component scales, those of Self Dysfunction were moderately (mid-0.40s) to moderately strongly (low-0.60s) intercorrelated, and those for Interpersonal Dysfunction correlated 0.46, in both cases showing an appropriate level of convergent validity for components of a broader construct (although it is important to remember that, different from the AMPD, they do not represent formal facets). However, Relationship Difficulties had essentially the same average correlation with the Self Dysfunction component scales (0.44) as it did with its counterpart, Dysfunctional Engagement (0.46), whereas the latter was largely independent of Self Dysfunction (average *r* = 0.11). Inspection of the item content suggests that this may be because Relationship Difficulties assesses various aspects of self-other interactions (e.g., conflicts, differences of opinion or in perspectives, becoming and being close to others), whereas Dysfunctional Engagement is focused on one’s general interest in having—and desire for being in—relationships. Thus Relationship Difficulties focuses more on “who one is” or “what one(self) is like in relationships,” thereby leading to a higher correlation with Self Dysfunction.

#### Convergent and Discriminant Validity of PD-Trait Scales

Trait-trait correlations at the domain level were also quite uneven, ranging in absolute value from 0.04 to 0.58, with an overall mean *r* of 0.30 (median *r* = 0.26). The three strongest correlations all involved NA, which correlated moderately strongly (*r* = 0.44 to 0.57) with all but DSL (*r* = 0.12), whereas the other trait-domain scale correlations were all negligible to moderate (range = 0.04 to 0.37; mean = 0.20). Thus, the trait scales were well-differentiated, for the most part, but the overlap of NA with three of the four other traits, and particularly DET warrants further investigation.

One other trait domain did not behave as expected: DSN. Most importantly, its two components related only moderately (*r* = 0.33) and, given this correlation, it is not surprising that the two components had different correlational patterns with other trait components: Distractibility correlated moderately strongly to strongly with two NA components, whereas Reckless Impulsivity correlated moderately strongly with the two Dissocial components. These results bring to mind the situation that existed with regard to trait impulsivity in the 1990s and early 2000s (e.g.,. Whiteside and Lynam, 2001; Whiteside et al., 2005; Sharma et al., 2013, 2014). Previously, impulsivity had been conceptualized as a single construct, even though research repeatedly showed that the term was used to characterize a heterogeneous set of dimensions. Whiteside and Lynam (2001) offered a conceptualization of “impulsive behaviors,” with distinct motivational factors underlying each of several groups.

The PID-5 literature also provides considerable support for the notion that Disinhibition may similarly characterize at least two quasi-independent aspects, one each reflecting more internalizing (e.g., difficulty concentrating) versus more externalizing (e.g., acting without consideration of consequences) forms of personality pathology. For example, PID-5 Disinhibition, particularly Distractibility, has been shown to correlate as strongly with NA as with FFM (low) Conscientiousness (e.g., Watson et al., 2013; Watson and Clark, 2020), to load on the NA/Neuroticism factor in both three-factor (Watson et al., 2013) and FFM (Thomas et al., 2013; Watson and Clark, 2020) frameworks, to correlate ≥ 0.40 with all six NEO-PI-3 (McCrae et al., 2005) facets (Watson and Clark, 2020), to correlate more strongly with PSY-5 Negative Emotionality than Disconstraint (Anderson et al., 2013), and to correlate with multiple scales of the Personality Assessment Inventory (PAI; Morey, 2007) that assess NA-related constructs. Despite the considerable support for splitting Distractibility off from Disinhibition—or at least considering it an interstitial dimension between Disinhibition and NA, the idea has not yet taken hold in the literature on the structure of maladaptive traits. Perhaps its time has come.

#### PD-severity–Trait Overlap

Having considered the convergent/discriminant patterns within each of the two major aspects of *ICD-11* PD—PD-severity and its trait qualifiers—we turn now to the important issue of PD-severity–trait overlap. The joint factor analysis of PD-severity and trait scales revealed considerable overlap of these two domains. The first two factors both had strong loadings from both PD-severity and trait component scales, with Self Dysfunction and NA dominating the first factor with a notable contribution from DSN Distractibility, whereas the second factor was marked most strongly by DET and Interpersonal Dysfunction components, followed by those of Anankastia, plus NA Mistrust. Finally, the third factor, in contrast to the first two, was essentially a pure trait factor, marked by both DSL components plus DSN Reckless Impulsivity, albeit with a modest cross-loading by PD-severity component Relationship Difficulties.

That Mistrust loads on the Internalizing Interpersonal Dysfunction factor makes sense because at the trait component level, Mistrust correlates more strongly with the two DET components than with the other two NA components (0.50 vs. 0.44). Although this difference is not statistically significant, it apparently is large enough to “pull Mistrust away” from loading with the other NA facets, suggesting that Mistrust may be an interstitial dimension. Support for this notion can be found in the PID-5 literature. For example, PID-5 Suspiciousness correlated at roughly the same magnitude with Neuroticism and Agreeableness factor scores in both Watson et al. (2013); 0.42 and −0.48, respectively) and Watson and Clark (2020); 0.51 and −0.53, respectively); loaded comparably on N, E, and low A when factored with either the NEO-PI-3 (0.35, 0.30, and 0.30, respectively; De Fruyt et al., 2013) or the Five-Factor Model Rating Form (Mullins-Sweatt et al., 2006; 0.30, 0.25, and 0.30, respectively; Thomas et al., 2013); and equally with the Negative Emotionality and Psychoticism scales of the PSY-5 (Harkness et al., 1995) (0.40 and 0.41, respectively, Anderson et al., 2013).

The most important point to discuss regarding this factor analysis is the intertwining of the PD-severity and trait scales, which warrants close consideration. One approach to understanding the considerable overlap between the PD-severity and trait domains would be to review the items of the scales that were particularly strongly correlated (and thus driving the factor structure) to determine whether they appropriately reflect their target constructs—that is, respectively, *functioning*—the “doing” aspect of personality—and *traits*, the “being” aspect of personality. That is, perhaps the scales’ items are not properly placed, are not clearly written, or reflect functioning-trait blends, such that the scales are inadvertently interstitial.

Relatedly, we might ask whether one (or both) of the strongly correlated components appropriately reflect aspects of the domain in which they currently are placed. For example, Self Dysfunction’s “Low Self-worth” component scale correlated more strongly (> 0.70) with two NA-component scales than with other Self Dysfunction component scales (*r*s ranged 0.45 to 0.53), so might it be more appropriately conceptualized as reflecting primarily NA rather than Self Dysfunction and therefore better placed within the former rather than within the latter? This seems to be the basis for the conclusion that McCabe et al. (2021) drew when they co-factored the Identity scales of various existing measures of AMPD Criterion A with traits and found that the Identity scales invariably marked the NA factor. Specifically, they stated that their study “suggests that the Criterion A self-identity scale can be understood as a maladaptive variant of FFM neuroticism” (p. 826).

A third possibility is to consider these results from a theoretical perspective and ask whether there may be certain inherently strong connections between PD severity and traits that are accurately reflected by these scales. Of note, the *ICD-11* description of PD severity includes “self-worth” among the “aspects of the self” that can manifest “problems in functioning” and the description of NA includes “low self-esteem” (World Health Organization, 2020c). Due to this shared quality, it is not surprising to find that Self Dysfunction and NA are strongly correlated. Digging down into the components of these domains, their descriptions have similar elements: The CDDG for PD-severity include “ability to maintain an overall positive and stable sense of self-worth” whereas those for NA include “exhibit low self-esteem and self-confidence.” Thus, perhaps self-worth/self-esteem and other such correlated constructs are truly interstitial, such that items written to reflect them in different domains will naturally be correlated and jointly determinant of a factor; in other words, perhaps self-worth is an inherently interstitial construct that links Self Dysfunction with NA. Put yet a fourth way, perhaps “being” and “doing” are themselves interconnected, such that certain PD-severity and trait components reflect *both* severity *and* trait variance to a considerable degree. In other words, perhaps it is better to conceptualize these components as reflecting “general PD pathology” rather than *either* PD-severity *or* trait variance.

Another example of the same idea can be found for interpersonal dysfunction and trait DET: the *ICD-11* CDDG description of (1) PD-severity includes “interpersonal dysfunction [in the] ability to develop and maintain close and mutually satisfying relationships” and (2) the trait qualifier Detachment states that its core feature “is the tendency to maintain interpersonal distance (social detachment) and emotional distance (emotional detachment)” (World Health Organization, 2020a). Given the near impossibility of developing close, satisfying relationships with others while maintaining interpersonal and emotional distance, Interpersonal Dysfunction and DET will necessarily correlate. And again, turning to the component level, Dysfunctional Engagement (e.g., not having satisfying relationships) and Social Detachment (e.g., not liking closeness with others) are also more strongly correlated with each other (*r* = 0.75) than either is with any other component scale.

The current data cannot adjudicate between the various possible interpretations described above; rather, they represent related theoretical interpretations and arguably are simply different ways of expressing the same basic idea. Our leaning is toward the last interpretation—the overlaps reflect general PD variance—as it seems the most parsimonious and does not require a dichotomous decision regarding whether certain scales assess dysfunction or trait variance. This perspective yields the conclusion that some characteristics of PD severity and traits are shared due to being general PD variance and others are at least somewhat more independent of each other (e.g., only one of the 12 correlations between the DSL and the PD-severity components reached 0.40—namely, DSL Low Empathy with Relationship Difficulties). Sharp et al. (2015) drew a similar conclusion (i.e., that personality pathology has both common and unique factors) in their bifactor analysis of *DSM-IV* PD criteria. This view does raise the question of why there is greater saturation of general PD pathology in some aspects of PD pathology than others, but answers to that question may not be simple, requiring more in-depth analyses at more specific levels of inquiry. This should not be surprising, given that we know already that the scope of some traits (e.g., NA) is broader than that of others (e.g., ANK); thus, it perhaps would be even more surprising if there were exactly the same degree of overlap among all PD-severity and trait constructs.

Conversely, it is surprising that Interpersonal Dysfunction did not correlate more strongly with DSL, given that its core feature is “disregard for the rights and feelings of others” (World Health Organization, 2020b), suggesting that more work may be needed on one or the other or both of these domains to reflect more clearly that interpersonal dysfunction necessarily includes disregard for the rights and feelings of others and that DSL necessarily involves interpersonal dysfunction, that is, that one cannot have good interpersonal functioning without having respect for the rights and feelings of others. For example, one description of PD-severity is “appropriateness of behavior responses to intense emotions and stressful circumstances (e.g., propensity to self-harm or violence” (World Health Organization, 2018, CDDG Draft Guidelines, p. 4) and this may not be adequately represented in our item pool. Alternatively, individuals high in DSL may have varying degrees of insight into the appropriateness of their behavioral response and, therefore, may not response “accurately” to relevant items if considered from the perspective of an objective observer.

In any case, the questions “How large is the ‘true’ degree of overlap between the PD-severity and PD-trait domains?” and “What is the nature of that overlap?” raise critical issues that have not yet been fully addressed. This is, in part, because most research on dimensional approaches to assessing personality pathology has not even included a separate measure of PD severity, and many of those that did used measures that were developed prior to the publication of the *DSM-5* AMPD or *ICD-11* (e.g., Berghuis et al., 2014). Further, to our knowledge, the current measure is the first for which PD-severity and trait scales have been co-developed. This is critical because separate development impedes the ability to consider such important questions as whether various qualities should be considered aspects of dysfunction severity, of traits, of both, or of a more general factor than either alone.

#### Limitations

The discussion above raises questions about the structure of the PD domain in the current study, including why the PD-trait analyses did not yield five factors and, relatedly, why the joint PD-severity and trait analyses did not yield at least five factors (i.e., five trait-based factors with Self- and Interpersonal Pathology component scales loading on those factors marked by the trait components with which they correlate).

One reason may be that, unlike the AMPD, the *ICD-11* PD-severity and traits are not formally faceted systems. For example, the AMPD PD-traits has 25 facets forming five domains, whereas the *ICD-11* PD-traits CDDG has 16 facet-like components (see Supplementary Table 1), and our preliminary analyses further reduced that number to 11 (see Table 1). There are several possible reasons for this and we highlight two of them here. First, in our attempt to limit the number of items administered, we may have “over-culled” the item pool, such that the pool that was administered had too few items for the various lower order components of the traits to form a robust set of constructs, thereby forcing them to form fewer, broader components. For example, per the CDDG description, we considered DET to have two main components—Social Detachment and Emotional Detachment—each of which had several subcomponents for which we wrote items, pruning these to the best 3-5 exemplars of each for administration. Had we retained more items per subcomponent, perhaps a more elaborated structure would have emerged. This limitation exists across domains, with only Self Dysfunction and NA ending up with more than two components.

Second, our criteria for retaining items at the data-analytic phase may have been too stringent, such that a more comprehensive, even if less clean, solution would have emerged had we been more lenient. For example, with very few exceptions, we used a 0.35 cut point for retaining items on a factor, and removed items with non-negligible cross-loadings. In some cases this meant eliminating too many items in a subfacet to retain it as such, resulting in a cleaner but less comprehensive structure. These decisions were made, in part, because we were mindful of reducing patient burden (i.e., being asked to complete a lengthy inventory), but this may have had unintended and unwanted structural consequences. Thus, just as the *ICD-11* has different versions for basic primary care settings, particularly in low- and middle-income countries, for use in mental-health-care settings, and for research, it is worth considering developing different versions of this measure of the *ICD-11* personality disorder model.

Another limitation of the current study is that it is based on a single online community sample of mostly white (> 90%) adults living in English-speaking countries, at least 75% of whom had been or were in mental-health treatment, and thus has not been cross-validated and also has somewhat limited generalizability. After revising the scales along the lines discussed above (e.g., using less stringent criteria for excluding items in an attempt to create a broader structure), it will be important to collect more data in a diversity of samples. Relatedly, the fact that the article reports on preliminary scales limits its contribution. In addition, the lack of external validity data also needs to be addressed in future research.

When the measure has been cross-validated, both in terms of its psychometric and structural properties and its convergent and discriminant validity with other measures of personality pathology, it will be important to translate it into multiple languages for international use, which we anticipated from the outset, so items were written with “translatability” in mind. These types of studies are in the planning stages.

## Conclusion

Despite these limitations, we believe that the current measure development project has promise for assessing the new *ICD-11* Personality Disorder model. Given the considerable differences between this model and those in previous *ICD* editions, having an instrument that can be used clinically as well as in research on the new model will be of considerable utility.

In sum, the *ICD-11* PD model represents a significant, one might say revolutionary, change in the conceptualization of personality pathology in two major ways. The first is the change from a set of discrete categories to a fully dimensional perspective. The second change is related yet importantly distinct, and that is to thinking of personality pathology as having two components. The first core and fundamental component is impairment in personality functioning—one might say in one’s personhood itself—that is, a general failure to mature adaptively and to develop the capacity to live successfully in one’s world. The second component, again related yet importantly distinct, is the more specific ways in which personality impairment is manifest, that is, an individual’s basic maladaptive-range personality traits. Each of these components contributes, in overlapping yet also distinct ways, to individuals’ specific patterns of emotional experience and expression, cognitive processes and beliefs, and behavioral manifestations of their personality. Like any conceptualization at its outset, the *ICD-11* PD model is a theory that needs to be tested empirically. To do so, we must be able to measure the components of the theory, and this project’s aim is to develop a measurement method specifically for that purpose.

## Author’s Note

YK is now at Concordia University in Edmonton, Canada. HFLA is now at Brown University. GSG is now at the University of Cambridge.

## Data Availability Statement

The dataset presented in this article are available only upon request for the purpose of verifying that the results reported are veridical. Requests to access the dataset should be directed to LAC, la.clark@nd.edu.

## Ethics Statement

The studies involving human participants were reviewed and approved by University of Notre Dame Institutional Review Board. The patients/participants provided their written informed consent to participate in this study.

## Author Contributions

LAC participated in data cleaning, analyzed the data, and wrote the first draft of the manuscript. SK wrote the initial draft of the IRB proposal and oversaw the data cleaning. DW participated in data cleaning, and reviewed and edited both the IRB proposal and the manuscript. ACE, HFLA, and GSG helped edit the manuscript. All authors participated in item writing and item selection.

## Conflict of Interest

The authors declare that the research was conducted in the absence of any commercial or financial relationships that could be construed as a potential conflict of interest.

## Footnotes

1 The copy of the CDDG to which the authors have access is labeled “Internal WHO Document for research use only; not for citation or distribution.” Thus, our descriptions of it follow the spirit of that statement.

2 It is important to note that our measure is not an official WHO measure. Moreover, given its preliminary status, we have not named it, but will do so when we finalize it.

3 One team member left the project midway and so is not included among the authors. We thank Sierria Gillon for her contributions.

4 Using the absolute value of the coefficients, as the degree rather than direction of overlap is what matters in computing discriminant validity.

5 The 0.46–0.33 comparison is not statistically significant if Bonferroni corrected for the total of 9 correlational comparisons we report in this article.
