# Supplementary material for: Preliminary Scales for ICD-11 Personality Disorder: Self and Interpersonal Dysfunction Plus Five Personality Disorder Trait Domains
Source: Front Psychol. 2021 Jul 12;12:668724. doi: 10.3389/fpsyg.2021.668724 (PMC8311289; doi:10.3389/fpsyg.2021.668724)
Supplement: Supplementary file 1 [file Table_1.pdf]

Supplemental Table 1

A Priori Rational Domains and Components/ Subcomponents of the ICD-11 Personality Disorder Model: Outline Derived from the Clinical Description and Diagnostic Guidelines

Domain	Components
	Core Personality Dysfunction[a]
Self Dysfunction	Disturbance in stability/ coherence of one's sense of identity
	Sense of self variable/inconsistent or overly rigid/fixed
	Low ability to maintain a positive sense of self-worth
	Low accuracy of one's view of one's characteristics, strengths and weaknesses (overly positive or negative)
	Low capacity for self-direction (low ability to plan, choose, and implement appropriate goals)
Interpersonal Dysfunction	Disturbance in multiple types of interpersonal relationships
	Low interest in engaging in interpersonal relationships
	Dysfunctional interest in interpersonal relationships (e.g., desperate need to have and hold relationships)
	Low ability to understand and appreciate others' perspectives
	Low ability to develop and maintain close relationships
	Low ability to manage conflict in relationships
	Personality Disorder Trait Qualifiers
Negative Affectivity	Experience of a broad range of negative emotions frequently and out of proportion to the situation
	Anxiety/ worry/ fear
	Depression/ vulnerability
	Anger/ hostility
	Guilt/ shame
	Emotional lability and poor emotion regulation
	Overreactivity to negative cognitions and external events
	Rumination of flaws, past mistakes, future problems
	Low frustration tolerance
	Emotional fluctuation or simultaneous mixed emotions
	Difficulty self-soothing
	Negativistic attitudes
	Responds negatively to advice and suggestions
	Pessimism/ hopelessness
	Resourcelessness/ helplessness
	Low self-esteem /self-confidence
	Avoidance of things judged to be too difficult
	Dependency
	Envy
	Suicidality

(table continues)

Supplemental Table 1 (cont.)

Domain	Components
(Negative Affectivity, cont.)	Mistrustfulness
	Suspiciousness./ distrustful of others' intentions
	Easily slighted or insulted
	Holds grudges
	Bitterness/ cynicism
Detachment	Social detachment
	Avoidance of social interactions, contact, and situations
	Avoidance of intimacy/ intimate relationships
	Lack of friendships or even acquaintances
	Lack of interest in sexual relations
	Lack of enjoyment in social interactions
	Emotional detachment
	Reserve, aloofness
	Keep to themselves even in social situations
	Limited emotion expression, both verbally and non-verbally
	Non-reactive to negative or positive events
	Limited emotional experience, lack of feeling(s)
	Limited capacity for enjoyment
Dissociality	Self-centeredness
	Sense of entitlement (i.e., preferentially deserve what they want without explanation or justification)
	Overly positive view of self (e.g., have admirable qualities, outstanding accomplishments, greatness) →
	Expectations of admiration
	Attention seeking, with negative behavior absent attention
	Focused on own needs, desires, or comfort
	Oblivious to others' needs, desires, or comfort
	Low empathy
	Lack of caring; callousness to others' suffering; in the extreme, take pleasure in inflicting pain/ harm
	Manipulativeness/ deceitfulness
	Exploitative for personal gain
	Aggressiveness/ meanness
	Ruthlessness in goal-seeking
	Unprovoked violence
Disinhibition	Impulsivity
	Rash behavior, with no regard for consequences, risk level
	Difficulty delaying gratification
	Pursuit of immediate pleasure
	Distractibility
	Difficulty sustaining attention
	Quickly bored/ frustrated by tedious/ difficult tasks
	Easily attracted by extraneous stimuli
	Scan environment for attractive alternatives

(table continues)

Supplemental Table 1 (cont.)

Domain	Components
(Disinhibition, cont.)	Irresponsibility, unreliability, low sense of accountabilitty
	Failure to meet expectations in completeness, timeliness
	Lack of follow-through on commitments, promises
	Late to appointments/ meetings
	Recklessness/ low cautiousness
	Engage in activities that put self and others in danger
	Overestimate own abilities and fail to consider safety risks
	Low planning/ preference for spontaneity
	Prefer having option to pursue attractive opportunities
	Low attention to goal setting
	Failure to follow through on plans
	Failure to meet goals
Anankastia	Perfectionism, rigidly expected from self and others
	Hyperconcern with rules, obligations, morality
	Scrupulous attention to detail
	Hyperemphasis on systems, routines, and schedules
	Hyperemphasis organization, orderliness, neatness
	Perseveration due to pursuit of perfection
	Clear, detailed personal ideas of perfection applied to all
	Emotional and behavioral constraint (both self and others)
	Rigidly controlled emotional expression
	Disapprove of others' displays of emotion
	Stubbornness, inflexibility,
	Averse to risk and uncertainty (due to lack of control)
	Difficulty decision making, due to hyperdeliberativeness

[a]Core personality dysfunction has emotional, cognitive, and behavioural manifestations. For example, emotional manifestations include restricted or extreme range of, or inappropriateness of, emotional experience and expression; under- or overreactivity, over- or underawareness of emotions. Cognitive manifestations include, for example, inaccuracy of situational and/or interpersonal appraisals; inappropriate decision-making, particular in situations of uncertainty; and/or highly unstable or overly rigid beliefs systems. Behavioural manifestations include, for example, inappropriate modulation of behavior based on the situation and potential consequences, and inappropriate behavioural responses to intense emotions and stressful situations.

Supplemental Table 2

Existing measures of the ICD-11 Personality Disorder Model Based on the PID-5

Bach et al. (2017)[a]<br>Name: None<br>Selected PID-5 facets<br>143 items – 16 facets<br>(Items per facet)	Sellbom et al. (2020)[a]<br>Name: None<br>Selected PID-5 facets<br>158 items – 18 facets<br>(Items per facet)	Kerber et al. (2020)<br>Name: PID-5-BF+[b]<br>Selected PID-5 items<br>34 items – 17 facets<br>Items per facet: 2	Bach et al. (2020)<br>Name: Modified PID-5-BF+[c]<br>Selected PID-5 items<br>36 items – 18 facets<br>Items per facet: 2	Bach & El Abiddine (2020)<br>Name: None<br>PID-5-BF (APA, 2013)<br>25 items<br>1 item per facet unless noted
		**Negative Affectivity**		
Emotional lability (7)	Emotional lability (7)	Emotional lability	Emotional lability	Emotional lability
Anxiousness (9)	Anxiousness (9)	Anxiousness	Anxiousness	Anxiousness
		Separation Insecurity	Separation Insecurity	Separation Insecurity
Depressivity (14)	Depressivity (14)			Depressivity [d]
Hostility (10)	Hostility (10)			Hostility
	Suspiciousness[e] (7)			
		**Detachment**		
Withdrawal (10)	Withdrawal (10)	Withdrawal	Withdrawal	Withdrawal (2)
Intimacy Avoidance (6)	Intimacy Avoidance (6)	Intimacy Avoidance	Intimacy Avoidance	Intimacy Avoidance[d]
		Anhedonia	Anhedonia	Anhedonia[d]
Restricted Affectivity (7)	Restricted Affectivity (7)			
		**Dissociality (Antagonism)**		
Grandiosity (6)	Grandiosity (6)	Grandiosity	Grandiosity	Grandiosity[d]
Manipulativeness (5)	Manipulativeness (5)	Manipulativeness	Manipulativeness	Manipulativeness
		Deceitfulness	Deceitfulness	Deceitfulness
Callousness (14)	Callousness (14)			Callousness
	Attention Seeking[f] (8)			Attention Seeking[e]

(table continues)

Supplemental *Table 2 (cont.)*

Bach et al. (2017)[a] Name: None Selected PID-5 facets 143 items – 16 facets (Items per facet)	Sellbom et al. (2020)[a] Name: None Selected PID-5 facets 158 items – 18 facets (Items per facet)	Kerber et al. (2020) Name: PID-5-BF+[b] Selected PID-5 items 34 items – 17 facets Items per facet: 2	Bach et al. (2020) Name: Modified PID-5-BF+[c] Selected PID-5 items 36 items – 18 facets Items per facet: 2	Bach & El Abiddine (2020) Name: None PID-5-BF (APA, 2013) 25 items 1 item per facet unless noted
		**Disinhibition**		
Impulsivity (6)	Impulsivity (6)	Impulsivity	Impulsivity	Impulsivity (2)
Irresponsibility (7)	Irresponsibility (7)	Irresponsibility	Irresponsibility	Irresponsibility
Distractibility (9)	Distractibility (9)	Distractibility	Distractibility	Distractibility[d]
Risk-taking (14)	Risk-taking (14)			Risk-taking
		**Anankastia**		
Rigid Perfectionism (10)	Rigid Perfectionism (10)	Rigid Perfectionism	Perfectionism[g]	
Perseveration (9)	Perseveration (9)	Perseveration	Rigidity[g]	
			Orderliness[g]	

Note. ICD-11 = International Classification of Diseases, 11th Ed. (World Health Organization, 2020); PID-5 = Personality Inventory for *DSM-5*; BF+ = Brief Form Plus. The 4-item Submissiveness facet of the Alternative Model for Personality Disorders (AMPD) is not included in any of the above measures. The AMPD's Psychoticism Domain is not included because it is not part of the *ICD-11* PD model.
[a]When also assessing the AMPD, the entire 220-item PID-5 is administered and the *ICD-11* PD model is scored from the listed facets. [b]Not based on the American Psychiatric Association (APA)'s 25-item PID-5-BF. [c]Modified version of the PID-5-BF+, not the APA's PID-5-BF. [d]Translated item for this facet did not load > .35 on any factor. [e]Translated item for this facet loaded .45 on Negative Affectivity. [f]Included only in Sellbom et al. (2020) based on the final *ICD-11* model. [g]Bach et al. (2020) included three Anankastia facets based on the original 37-facet model of the AMPD. For Perfectionism, the two items selected by Kerber et al. (2020) items were used; for Rigidity, the only two items in the PID-5 item pool that tapped Rigidity were used; for Orderliness, four possible PID-5 items were identified and the two with the strongest psychometric characteristics were selected.

Supplemental Table 3

One-Factor Principal Axis Factor Analysis of ICD-11 PD-Severity Component Scales

Domain	Component	Personality Pathology
SP	Identity	**.80**
SP	Low Self-directedness	**.72**
IP	Relationship Difficulties	**.67**
SP	Low Self-accuracy	**.66**
SP	Low Self-worth	**.65**
IP	Dysfunctional engagement	.28

Note. *N* = 383 community adults. *ICD-11* = International Classification of Diseases, 11th Ed. (World Health Organization, 2020); PD= Personality Disorder; SP = Self Dysfunction; IP = Interpersonal Dysfunction.

Factor loadings ≥ .40 are **bolded**; those < .40 and ≥ .30 are *italicized.*

Supplemental Table 4

One-Factor Principal Axis Factor Analysis of ICD-11 PD-Trait Component Scales

Domain	Component	PD Trait Pathology
NA	Negative Outlook	**.80**
DET	Social Detachment	**.74**
NA	Emotional Lability	**.67**
DET	Emotional Detachment	**.67**
NA	Mistrust	**.63**
ANK	Hypercontrol	**.62**
DSN	Distractibility	**.59**
ANK	Perfectionism	.25
DSN	Reckless Impulsivity	.10
DSL	Low Empathy	*.35*
DSL	Entitled Superiority	-.04

Note. *N* = 383 community adults. *ICD-11* = International Classification of Diseases, 11th Ed. (World Health Organization, 2020); PD= Personality Disorder; NA = Negative Affectivity; DET = Detachment; ANK = Anankastia; DSN = Disinhibition; DSL = Dissociality.

Factor loadings ≥ .40 are **bolded**; those < .40 and ≥ .30 are *italicized.*

Supplemental Table 5

One-Factor Principal Axis Factor Analysis of ICD-11 PD-Severity and Trait Component Scales

Domain	Facet	Personality Pathology Severity and Traits
NA	Negative Outlook	**.87**
ID	Relationship Difficulties	**.77**
SD	Low Self-directedness	**.74**
NA	Emotional Lability	**.72**
DET	Social Detachment	**.71**
SD	Low Self-worth	**.71**
SD	Identity Problems	**.70**
DSN	Distractibility	**.68**
DET	Emotional Detachment	**.64**
NA	Mistrust	**.60**
SD	Low Self-accuracy	**.54**
ANK	Hypercontrol	**.54**
ID	Dysfunctional Engagement	**.45**
DSN	Reckless Impulsivity	.17
DSL	Low Empathy	*.30*
DSL	Entitled Superiority	-.08
ANK	Perfectionism	.16

Note. *N* = 383 community adults. *ICD-11* = International Classification of Diseases, 11th Ed. (World Health Organization, 2020); PD= Personality Disorder; NA = Negative Affectivity; ID = Interpersonal Dysfunction; SD = Self Dysfunction; DET = Detachment; DSN = Disinhibition; PP = Perseverance and Planning; ANK = Anankastia; DSL = Dissociality.

Factor loadings ≥ .40 are **bolded**; those < .40 and ≥ .30 are *italicized.*

Supplemental Table 6

Promax-Rotated Two-Factor Principal Axis Factor Analyses of ICD-11 PD Trait Component Scales

Domain	Facet	Internalizing	Externalizing
NA	Negative Outlook	**.80**	.02
DET	Social Detachment	**.76**	-.10
ANK	Hypercontrol	**.69**	-.29
NA	Emotional Lability	**.67**	.01
DET	Emotional Detachment	**.66**	.09
NA	Mistrust	**.61**	.12
DSN	Distractibility	**.55**	.24
ANK	Perfectionism	.27	-.11
DSN	Reckless Impulsivity	-.05	**.77**
DSL	Low Empathy	.21	**.72**
DSL	Entitled Superiority	-.16	**.58**

Note. *N* = 383 community adults. *ICD-11* = International Classification of Diseases, 11th Ed. (World Health Organization, 2020); PD= Personality Disorder; NA = Negative Affectivity; DET = Detachment; ANK = Anankastia; DSN = Disinhibition; PP = Perseverance and Planning; DSL = Dissociality.

Factor loadings $\geq$ .40 are **bolded**; those < .40 and $\geq$ .30 are *italicized.*

Supplemental Table 7

Promax-Rotated Two-Factor Principal Axis Factor Analyses of ICD-11 PD-Severity and Trait Component Scales

Domain	Facet	Internalizing	Externalizing
NA	Negative Outlook	**.88**	-.02
ID	Relationship Difficulties	**.75**	.19
SD	Low Self-worth	**.73**	*-.31*
SD	Low Self-directedness	**.73**	.09
DET	Social Detachment	**.73**	-.21
NA	Emotional Lability	**.73**	-.09
SD	Identity Problems	**.69**	.13
DSN	Distractibility	**.66**	.21
DET	Emotional Detachment	**.64**	.02
NA	Mistrust	**.59**	.04
ANK	Hypercontrol	**.57**	*-.38*
SP	Low Self-accuracy	**.53**	.17
IP	Dysfunctional engagement	**.46**	-.05
ANK	Perfectionism	.18	-.17
DSN	Reckless Impulsivity	.10	**.79**
DSL	Low Empathy	.25	**.66**
DSL	Entitled Superiority	-.13	**.55**

Note. *N* = 383 community adults. *ICD-11* = International Classification of Diseases, 11th Ed. (World Health Organization, 2020). PD= Personality Disorder, NA = Negative Affectivity, IP = Interpersonal Dysfunction, SP = Self Dysfunction, DET = Detachment, DSN = Disinhibition, PP = Perseverance and Planning, ANK = Anankastia, DSL = Dissociality.

Factor loadings ≥ .40 are **bolded**; those < .40 and ≥ .30 are *italicized.*

Supplemental Table 8

Promax-Rotated Four-Factor Principal Axis Factor Analyses of ICD-11 PD-Severity and Trait Component Scales

Domain	Facet	Internalizing Self Dysfunction	Internalizing IP Pathology	Externalizing Traits	Anankastia
SD	Low Self-directedness	**.83**	.15	-.10	-.28
NA	Negative Outlook	**.83**	.08	-.08	.14
SD	Identity Problems	**.78**	-.01	.05	.04
DSN	Distractibility	**.73**	.14	.07	-.23
SD	Low Self-accuracy	**.73**	-.12	.12	.02
SD	Low Self-worth	**.71**	-.09	-.24	*.34*
NA	Emotional Lability	**.62**	-.06	.04	**.42**
ID	Dysfunctional Engagement	-.21	**.92**	-.02	-.06
DET	Social Detachment	.06	**.86**	-.16	.06
DET	Emotional Detachment	.12	**.69**	.06	.02
ID	Relationship Difficulties	*.36*	*.39*	*.30*	.24
NA	Mistrust	.19	*.39*	.17	.26
DSL	Low Empathy	-.01	.28	**.74**	.03
DSL	Entitled Superiority	-.19	-.14	**.71**	.25
DSN	Reckless Impulsivity	*.33*	-.11	**.68**	-.29
ANK	Perfectionism	-.16	-.01	.13	**.67**
ANK	Hypercontrol	.16	.17	-.12	**.63**

Note. *N* = 383 community adults. *ICD-11* = International Classification of Diseases, 11th Ed. (World Health Organization, 2020). PD= Personality Disorder, NA = Negative Affectivity, IP = Interpersonal Dysfunction, SD = Self Dysfunction, DET = Detachment, DSN = Disinhibition, PP = Perseverance and Planning, ANK = Anankastia, DSL = Dissociality.

Factor loadings ≥ .40 are **bolded**; those < .40 and ≥ .30 are *italicized.*
